# Lnc2Cancer: a manually curated database of experimentally supported lncRNAs associated with various human cancers

**DOI:** 10.1093/nar/gkv1094

**Published:** 2015-10-19

**Authors:** Shangwei Ning, Jizhou Zhang, Peng Wang, Hui Zhi, Jianjian Wang, Yue Liu, Yue Gao, Maoni Guo, Ming Yue, Lihua Wang, Xia Li

**Affiliations:** 1 College of Bioinformatics Science and Technology, Harbin Medical University, Harbin 150081, China; 2 Department of Neurology, The Second Affiliated Hospital of Harbin Medical University, Harbin 150081, China

## Abstract

Lnc2Cancer (http://www.bio-bigdata.net/lnc2cancer) is a manually curated database of cancer-associated long non-coding RNAs (lncRNAs) with experimental support that aims to provide a high-quality and integrated resource for exploring lncRNA deregulation in various human cancers. LncRNAs represent a large category of functional RNA molecules that play a significant role in human cancers. A curated collection and summary of deregulated lncRNAs in cancer is essential to thoroughly understand the mechanisms and functions of lncRNAs. Here, we developed the Lnc2Cancer database, which contains 1057 manually curated associations between 531 lncRNAs and 86 human cancers. Each association includes lncRNA and cancer name, the lncRNA expression pattern, experimental techniques, a brief functional description, the original reference and additional annotation information. Lnc2Cancer provides a user-friendly interface to conveniently browse, retrieve and download data. Lnc2Cancer also offers a submission page for researchers to submit newly validated lncRNA-cancer associations. With the rapidly increasing interest in lncRNAs, Lnc2Cancer will significantly improve our understanding of lncRNA deregulation in cancer and has the potential to be a timely and valuable resource.

## INTRODUCTION

Cancer is a leading cause of death worldwide and has emerged as a major public health problem in many countries (1). Cancer is a complex disease involving multiple levels of alterations, including genetic, epigenetic and transcriptomic alterations (2). One of the most important tasks in cancer research is to understand the molecular mechanisms underlying these alterations.

In recent years, increasing evidence has suggested that a novel class of non-coding RNA, long non-coding RNA (lncRNA), is commonly altered at various stages of cancer progression (3,4). lncRNAs are a class of pervasively transcribed RNA molecules with a length of more than 200 nucleotides that do not encode proteins (5). Studies have indicated that lncRNAs play critical roles in a wide range of biological processes (6,7). Due to their functional significance, several databases have been developed to store lncRNA-related information. For example, NONCODE (8), lncRNAdb (9), LNCipedia (10) and LncRNAWiki (11) integrate lncRNA data obtained from different sources. ChIPBase (12) is focused on the transcriptional regulation of miRNAs and lncRNAs. DIANA-LncBase (13) identifies miRNA–lncRNA interactions. LncRNADisease (14), lncRNASNP (15) and LincSNP (16) contain different lncRNA and disease associations. These databases are crucial for deciphering lncRNA functions in human cancers. However, our knowledge of cancer-related lncRNAs remains limited. In particular, a public resource of high-quality curated cancer-associated lncRNAs remains unavailable.

Recent publications of large-scale cancer genomic datasets, such as The Cancer Genome Atlas (17), provide an opportunity to investigate cancer-related lncRNAs in a large sample (18,19). In addition to these large-scale studies, studies focused on individual or specific lncRNA in various cancers are rapidly emerging (20,21). Currently, accumulating evidence suggests that the deregulation of lncRNAs plays an important role in human cancers (22). However, these experimentally supported lncRNA-cancer associations are hidden in thousands of published studies. These fragmented and even inconsistent publications are an obstacle to characterizing lncRNA functions in cancer from a global view. In addition, several cancer-specialized databases have been published that focus on some important topics. For example, COSMIC (23) collects cancer genes and mutations, MethyCancer (24) and PubMeth (25) aim to investigate DNA methylation in cancer, and miRCancer (26) provides literature-supported microRNA and cancer associations; however, no resource is currently devoted to collecting the latest and experimentally supported lncRNA-cancer associations.

To address this gap, we developed a database, called Lnc2Cancer, to collect and integrate cancer-associated lncRNAs into a comprehensive resource. All lncRNA-cancer associations in the Lnc2Cancer are experimentally supported and manually curated from the published literature. The current version of Lnc2Cancer documents 1057 manually curated associations between 531 lncRNAs and 86 human cancers. We hope that this elaborate database specially designed for cancer and lncRNA could serve as an important catalyst for future research.

## DATA COLLECTION AND DATABASE CONTENT

To ensure the highest quality in the data collection process, all Lnc2Cancer entries were manually collected through several steps as previously described (27–29). First, we searched the PubMed database (30) with a list of keywords, such as ‘lncRNA cancer,’ ‘long non-coding RNA cancer,’ ‘lncRNA tumor,’ ‘long non-coding RNA tumor,’ ‘lncRNA neoplasm,’ and ‘long non-coding RNA neoplasm.’ We downloaded all published literature describing the associations between lncRNAs and human cancers. Second, we extracted experimentally supported lncRNA-cancer associations ‘by hand,’ that is, by manually curating from published papers. All selected studies were reviewed by at least two researchers. In this step, we retrieved the lncRNA and cancer name, sequence and positional information of the lncRNAs, experimental techniques (e.g., microarray, Northern blot, qRT-PCR), experimental samples (cell line and/or tissue), expression patterns of lncRNA (upregulated or downregulated), hyperlinks to the PubMed database (PubMed ID, year of publication, title of paper) and a brief functional description of lncRNA from the original studies. In particular, we have referred to previous studies and selected lncRNA-cancer associations for manual curation using strict criteria (31). We only collected high-quality associations with multiple lines of strong experimental evidence, experimentally confirmed by RNAi, *in vitro* knockdown, Western blot, qRT-PCR or luciferase reporter assays. Third, all selected studies were rechecked for the lncRNA names and cancers, and some names were replaced with official or recommended names. In this step, we also collected other names, including aliases and synonyms, for lncRNAs. To make the lncRNA names more complete and consistent with other databases, we have provided both the identifiers and links for the lncRNAs present in the Ensembl and RefSeq database. Finally, we used a standardized classification scheme, the International Classification of Diseases for Oncology, 3rd Edition (ICD-O-3), to annotate each cancer type.

After completing the above processes, more than 1500 published papers were systematically reviewed, and a total of 1057 associations between 531 lncRNAs and 86 human cancers were manually curated. Moreover, we provided useful links to other databases, including lncRNAs in GenBank, HGNC, lncRNAdb and NONCODE and cancers in the OMIM and COSMIC databases, among others. We also provided several links to web-based computational tool to annotate lncRNA functions in cancer, such as LncRNA2Function (32) and Co-LncRNA (33), which allows users to identify GO annotations and KEGG pathways that might be affected by single or multiple lncRNAs.

Finally, all data in Lnc2Cancer were stored and managed using MySQL (version 5.5.58). The web interfaces were built in JSP. The data processing programs were written in Java (version 1.6.0), and the web services were built using Apache Tomcat. The Lnc2Cancer database is freely available at http://www.bio-bigdata.net/lnc2cancer and http://www.bio-bigdata.com/lnc2cancer.

## USER INTERFACE

**Figure 1.**
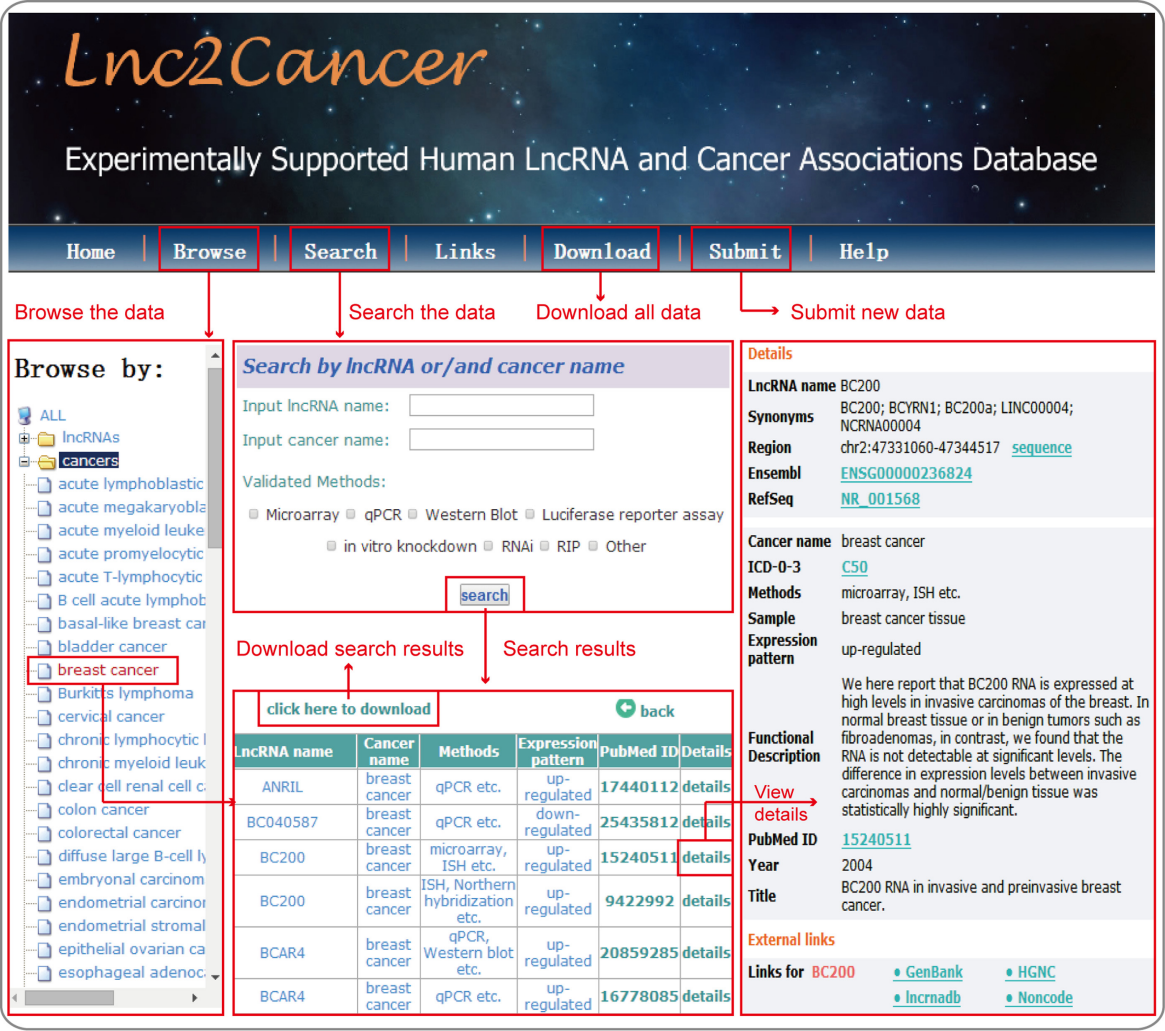
A schematic workflow of Lnc2Cancer.

Lnc2Cancer provides a user-friendly web interface that enables users to browse, search and retrieve all lncRNA-cancer associations in the database (Figure 1). In the ‘Browse’ page, users can browse Lnc2Cancer by clicking a specific lncRNA or cancer name, and a list of matched entries is returned. In the ‘Search’ page, Lnc2Cancer allows users to search by lncRNA name and alternative name, cancer name or both. Lnc2Cancer offers fuzzy keyword searching capabilities, facilitating searches by returning the closest possible matching records. Lnc2Cancer provides an option in the ‘Search’ page that allows users to filter associations by certain experimental methods. Lnc2Cancer also offers a submission page that enables researchers to submit novel experimentally supported lncRNA-cancer associations. Once approved by the submission review committee, the submitted record will be included in the update release. In addition, all data in the database can be downloaded in the ‘Download’ page, and a detailed tutorial showing users how to use Lnc2Cancer is available on the ‘Help’ page.

## FUTURE EXTENSIONS

More recently, high-throughput technologies, such as next-generation sequencing, have produced extensive data on cancer biology, and the number of validated cancer-associated lncRNAs will continue to increase in the future. These advances in research will provide the opportunity to further extend Lnc2Cancer. We will continue to manually curate newly validated lncRNA-cancer associations and update the database every 2 months. We will incorporate new tools and functional annotations as well as more data sources to improve the utility and content coverage of this database. In addition, several recent large-scale RNA-sequencing datasets have generated a large number of lncRNA-cancer associations (34). Although there is often no further strong experimental evidence to confirm these associations, they also have potential value for cancer study. We will develop novel methods to filter these associations in future studies.

## DISCUSSION AND CONCLUSION

Emerging evidence suggests that aberrant expression of lncRNAs plays a critical role in human cancers. Because most cancer-related lncRNAs are identified in independent studies that have been performed over a period of many years, a curated collection of these lncRNAs will provide researchers with a vital resource for cancer research. Currently, there are several databases that can provide lncRNA-cancer associations for researchers (Supplementary Table S1). For example, the lncRNASNP (15) and LincSNP (16) databases store a number of cancer-associated SNPs in human lncRNAs. However, these lncRNA-cancer associations were inferred by computational methods and without direct experimental evidence. To the best of our knowledge, only one database, LncRNADisease (14), partially addresses the needs of cancer research. However, in the current version of LncRNADisease, there are 492 cancer-related entries that include only 128 human lncRNAs and 55 cancer types, and there are no explicit descriptions of the experimental techniques to find these associations. Thus, we developed Lnc2Cancer, a cancer-specialized database that provides a comprehensive resource on lncRNA dysregulation in various human cancers.

In addition to including a greater number of lncRNA-cancer associations, Lnc2Cancer has several advanced features that distinguish it from previous studies. For example, by searching Lnc2Cancer using ‘HOTAIR,’ a well-known human lncRNA, we found that the expression of HOTAIR is ‘upregulated’ in almost all cancer types, and this finding is supported by multiple lines of strong experimental evidence. In addition, Lnc2Cancer provides functional information on lncRNAs in cancer. For example, by searching Lnc2Cancer using both ‘HOTAIR’ and ‘cervical cancer,’ Lnc2Cancer shows that high HOTAIR expression in cervical cancer is correlated with lymph node metastasis and reduced overall survival and that HOTAIR regulates the expression of vascular endothelial growth factor, matrix metalloproteinase-9 and epithelial-to-mesenchymal transition-related genes, which are important for cell motility and metastasis (35). Lnc2Cancer indicates that these functional roles of HOTAIR are supported both in cell lines (SiHa, HeLa, Caski) and in cervical cancer tissue, which may be especially useful for cancer specialists and biologists.

**Figure 2.**
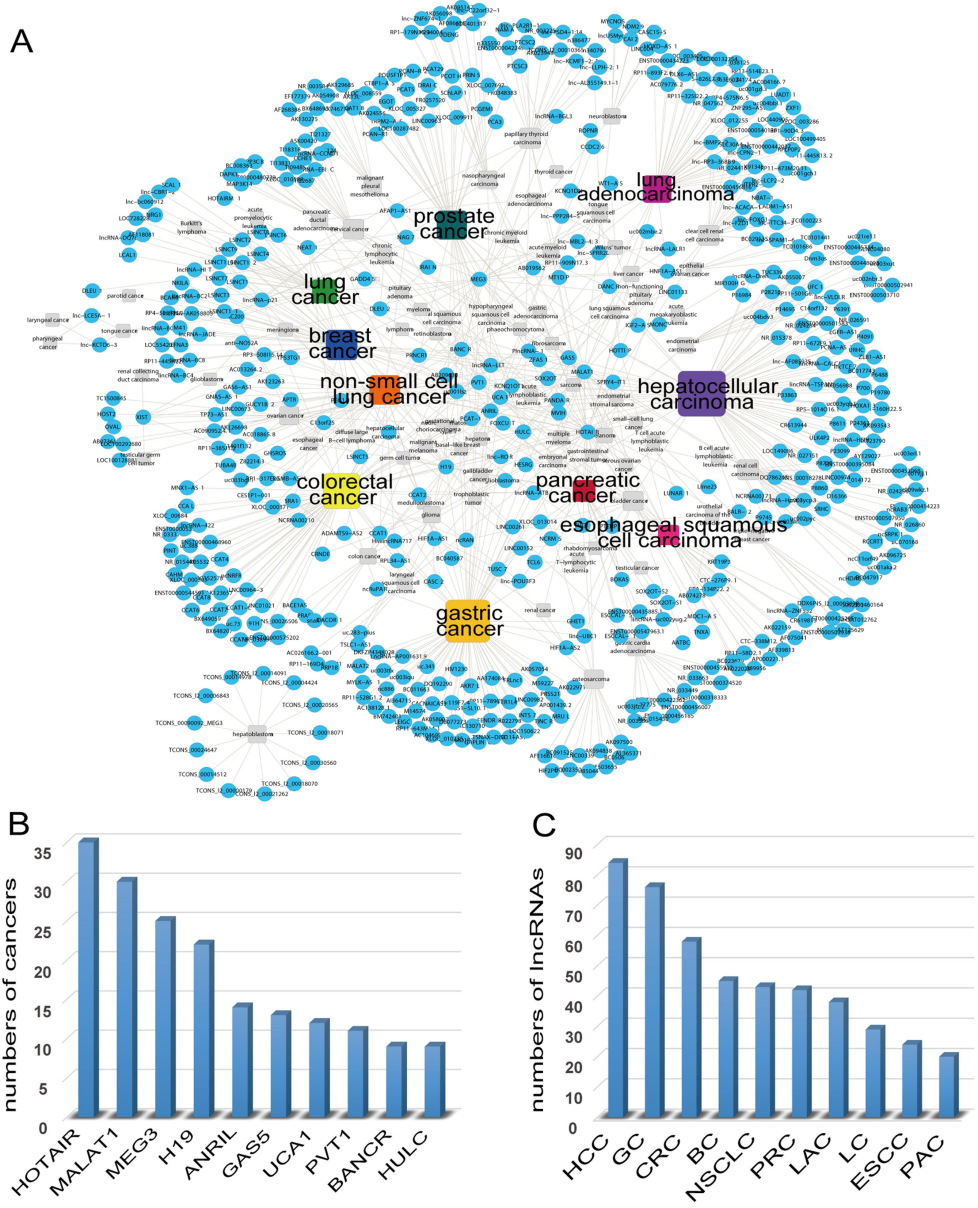
Network and distribution of cancers and lncRNAs in Lnc2Cancer. (**A**) Human lncRNA-cancer bipartite network. Circles and rectangles correspond to lncRNAs and cancers, respectively, and the lines correspond to experimentally supported associations. The size of the nodes corresponds to the nodes’ degree. The ten cancers with the highest connectivity are colored, whereas other cancers are shown in gray. Distribution of the ten lncRNAs (**B**) and cancers (**C**) with the highest connectivity in bipartite network. Abbreviations: HCC (hepatocellular carcinoma), GC (gastric cancer), CRC (colorectal cancer), BC (breast cancer), NSCLC (non-small-cell lung cancer), PRC (prostate cancer), LAC (lung adenocarcinoma), LC (lung cancer), ESCC (esophageal squamous-cell carcinoma), PAC (pancreatic cancer).

By analyzing the data from Lnc2Cancer, we could find some important principles behind a large, complex and integrated resource. We constructed an lncRNA-cancer bipartite network (Figure 2A) in which the nodes denote lncRNAs or cancers and the lines correspond to experimentally supported associations between lncRNAs and cancers. We also list the ten most highly connected nodes in this bipartite network, including lncRNAs and cancers (Figure 2B and C, respectively). Previous studies have shown that if a node has more links within a given network, then loss of this node would generally greatly impact network behavior (36). We found that the cancer with the highest connectivity is hepatocellular carcinoma, which is associated with 86 lncRNAs. For lncRNAs, HOTAIR is the most highly connected node, which is associated with 35 cancers, thus providing further evidence for the importance of this lncRNA in human cancers (37). In addition, we counted the number of published papers each year that reported lncRNA-cancer associations and found that the publications generally increase dramatically (Supplementary Figure S1). Especially from 2012 to 2014, the number of publications increased in an exponential manner, suggesting that research on lncRNA-cancer associations has become a hot topic in recent years, thus providing us with tremendous power to develop a special-purpose repository to document these valuable data.

In summary, Lnc2Cancer not only provides more than 1000 manually curated lncRNA-cancer associations with experimental support but also offers global insights into lncRNA functions in human cancers. This unique and cancer-specialized database created here will be useful for future research.
